# Preparation of CaCl_2_/MOF-303 composite and its dehumidification properties

**DOI:** 10.1039/d4ra08282f

**Published:** 2025-03-24

**Authors:** Ying Li, Jianzhe Li, Shumeng Yin, Xiaowen Shan, Bin Tao, Shiqiang Wang

**Affiliations:** a State Key Laboratory of Chemical Safety, SINOPEC Research Institute of Safety Engineering Co., Ltd Qingdao 266104 Shandong Province China liying.chemistry@outlook.com yinsm.qday@sinopec.com

## Abstract

A series of aluminium based Metal–Organic Framework (Al-MOF) composite adsorbents were prepared by impregnating moisture-sensitive CaCl_2_ with different relative contents into Al-MOF (MOF-303). The composite adsorbents were characterized by adsorption isotherm of N_2_, elemental analysis and scanning electron microscopy, and subjected to static and dynamic adsorption tests of water vapor, as well as cyclic adsorption and desorption tests. The results showed that with the addition of CaCl_2_, the high surface area of MOF-303 granules (1276 m^2^ g^−1^) dropped sharply to 588–683 m^2^ g^−1^. However, under the synergistic effect of physical adsorption and chemical adsorption, the purification effects of the composite adsorbents were significantly better than those of unmodified MOF-303, molecular sieves, and silica gel. The adsorption performance was correlated with the impregnation amount of CaCl_2_. As the CaCl_2_ content increased, the saturation adsorption capacity and breakthrough adsorption capacity of the composite adsorbents all showed a trend of first increasing and subsequently decreasing. The maximum water adsorption capacity of the CaCl_2_/MOF-303 composite was 1077 mg g^−1^. In addition, the regenerative rate of the CaCl_2_/MOF-303 composite was over 96.1% after fifty adsorption and desorption cycles of water, showing good desorption performance and excellent structural stability, which proved a broad application prospect in the field of dehumidification.

## Abstract 2

CaCl_2_/MOF-303 composites were prepared by impregnating CaCl_2_ into MOF-303, they showed large water adsorption capacity, high regenerative rate and excellent structural stability, proving a broad application prospect in the field of dehumidification.

## Graphical abstract

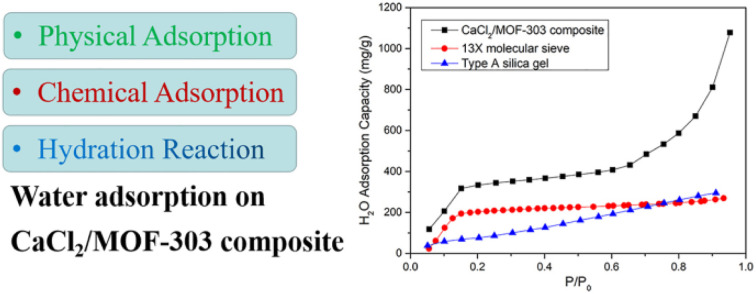

## 1 Introduction

In numerous chemical production processes, the dehydration of raw materials is essential to enhance reaction efficiency. For instance, in olefin polymerization and olefin disproportionation, removing moisture from olefin feedstocks can significantly extend catalyst lifetimes and improve overall process efficiency.^1,2^ Adsorption technology, known for its energy efficiency, high performance, simplicity, and low cost, has become a cornerstone in industrial applications such as gas adsorption, separation, and dehumidification.^3–6^ Among the common water-adsorbing materials, porous materials and non-porous inorganic compounds are frequently used. Molecular sieves, in particular, are favored for their strong polarity and excellent regeneration stability, making them widely used in the purification of various chemical feedstocks.^7–11^ However, their reliance on physical adsorption limits their water uptake capacity, necessitating frequent regeneration and increasing operational complexity and costs. Additionally, the large-scale adsorption beds or dehumidification equipment required for molecular sieve applications further restrict their practical use. Typical hygroscopic inorganic compounds such as MgSO_4_, MgCl_2_ and CaCl_2_ can adsorb large amounts of water at room temperature and return to their original state when heated.^12,13^ However, their strong hygroscopic nature leads to issues such as dry shrinkage and caking, rendering them unsuitable for the dehydration and adsorption processes of chemical feedstocks.

In recent years, the development of new dehumidifying materials has garnered significant attention. Among these, metal–organic frameworks (MOFs) have emerged as promising candidates due to their high specific surface area, large porosity, structural diversity, and excellent hygroscopicity.^14–25^ Their ability to adsorb water surpasses that of traditional porous materials such as molecular sieves and silica gel. In various MOFs used for water adsorption, the N(H) group of the PZDC^2−^ (1*H*-pyrazol-3,5-dicarboxylate) connector in MOF-303 [Al(OH)(PZDC)] is the main adsorption site of water molecules, and the strength of this interaction is conducive to the adsorption of water molecules.^26–29^ Moreover, MOF-303 demonstrates excellent multi-cycle stability, lacks harmful components, and features a simple, environmentally friendly synthesis route, making it highly attractive for dehumidification applications.

Despite extensive research on MOF-based dehumidification, there remains a need to develop porous materials with even higher water adsorption capacities. One promising strategy is to prepare composite adsorbents by incorporating hygroscopic inorganic compounds into the pores of MOFs. This approach not only enhances the adsorption capacity of the porous adsorbents but also addresses issues such as caking and agglomeration of inorganic salts after water adsorption. Additionally, powder adsorbents often suffer from high pressure drop, poor heat conduction, and susceptibility to external environmental influences, further complicating their practical application. Therefore, it is crucial to directly prepare and investigate the water absorption properties of granular adsorbents.

**Fig. 1:**
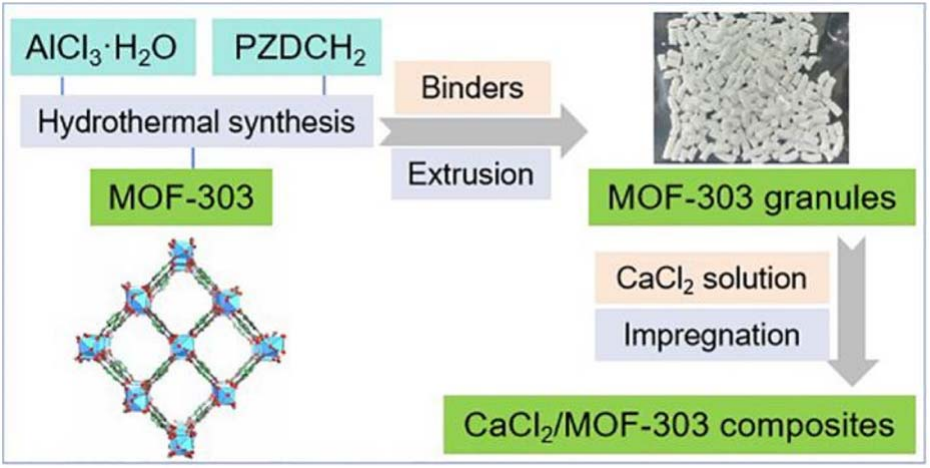
The preparation diagram of the CaCl_2_/MOF-303 composite.

In this study, we introduced a novel approach to address these challenges by preparing a series of CaCl_2_/MOF-303 granular composite adsorbents *via* an impregnation method (Fig. 1). Through comprehensive characterization techniques, including N_2_ physical adsorption, elemental analysis, and scanning electron microscopy, we systematically investigated the loading capacity of CaCl_2_ within the composite, the changes in the microstructure of the adsorbents, and their impact on both static and dynamic water adsorption properties. Furthermore, we evaluated the cyclic adsorption–desorption performance of the composite, highlighting its potential for practical dehumidification applications. This work represents a significant advancement in the development of high-performance composite adsorbents, offering a balanced combination of enhanced water uptake capacity and improved structural stability.

## 2 Experimental

### 2.1 Experimental materials

All chemicals and regents were analytical grade in purity and used without further processing. Aluminum chloride hexahydrate (98.0%), 3,5-pyrazolecarboxylic acid monohydrate (97.0%), methyl cellulose (99.0%, 40 000 mPa s), NaOH (99.0%), calcium chloride (96.0%), and anhydrous ethanol (99.5%) were all purchased from Macklin Biochemical Co. Ltd (Shanghai, China).

### 2.2 Preparation of MOF-303 powder

MOF-303 was prepared using the method described in ref. 30. Aluminum chloride hexahydrate (10.4 g, 43.08 mmol) and 3,5-pyrazole dicarboxylic acid monohydrate (7.5 g, 43.08 mol) were dissolved in a 1 L glass reaction bottle containing 720 ml water, respectively, and the mixed solution was stirred evenly at room temperature. NaOH (2.6 g, 65 mmol) was dissolved in 30 ml water, then the solution was stirred evenly and slowly added to the mixed solution of aluminum chloride and 3,5-pyrazole dicarboxylic acid. Subsequently, the mixed solution was heated at 373 K for 24 h, cooled to room temperature, and filtered to separate the white solid. The solids were washed three times with deionized water and ethanol to remove the unreacted raw materials and impurities. The air-dried solids were transferred to a vacuum oven and heated at 373 K for 24 h to obtain MOF-303 powders.

### 2.3 Preparation of MOF-303 granules

Al-MOF powder (2.85 g) and methylcellulose (0.15 g) were added to a 100 ml beaker. After the solid powders were evenly mixed, the mixture of deionized water/ethanol (1 : 1) was slowly added while stirring, and a total of 4.5 ml of ionized water/ethanol mixture was added. After the solid and liquid were evenly mixed, the mud was squeezed into long strips with a 10 ml syringe and fully dried. The strips were cut into 2–5 mm cylindrical granules. After air-dried, the MOF-303 granules were heated in a vacuum oven at 373 K for 8 h.

### 2.4 Preparation of CaCl_2_/MOF-303 composite adsorbents

Impregnation method: aqueous solutions of CaCl_2_ with concentrations of 10%, 20%, 30% and 40% w(CaCl_2_) were prepared, and MOF-303 granules were immersed in excess of the above solutions, respectively. After immersion for 4 h, the CaCl_2_ solution was filtered out and the surface residual CaCl_2_ on the surface of the granules was washed away with deionized water. Subsequently, the samples prepared above were dried in a muffle furnace at 423 K until the mass no longer decreased. The MOF-303 which was not loaded with CaCl_2_ was labeled as A0, and the samples obtained by impregnating CaCl_2_ solution with different concentrations were named as CaCl_2_/MOF-303 (A1), CaCl_2_/MOF-303 (A2), CaCl_2_/MOF-303 (A3), CaCl_2_/MOF-303 (A4), and abbreviated as A1, A2, A3 and A4, respectively.

Physical mixing method: in order to compare the properties of CaCl_2_/MOF-303 composites prepared by impregnation method, MOF-303 powder was physically mixed with CaCl_2_ powder according to the content of CaCl_2_ in CaCl_2_/MOF-303 composites synthesized by impregnation method.

### 2.5 Characterization

The specific surface area and pore size of the samples were analyzed by BSD-PM2 specific surface area and pore size analyzer. Before the test, the samples were placed in a vacuum at 423 K for 6 h activation pretreatment, and then N_2_ adsorption isotherm test was carried out at 77 K. The BET and Langmuir specific surface area of the materials were calculated by the BET and Langmuir equations respectively, the *t*-plot model was used to analyze the pore volume of the micropore (<2 nm), and the H–K equation was used to calculate the pore size of the micropore range.

Inductively coupled plasma emission spectroscopy (ICP-OES) was used to analyze the calcium content of composites. In order for it to dissolve completely, the samples were treated with a mixture of sulfuric and nitric acid at 573 K. To evaluate the reproducibility of the analysis, the calcium content of each compound was measured twice on the same batch of samples at different times, and the average of the two results was taken. Chemical properties of the MOF-303, and CaCl_2_/MOF-303 and CaCl_2_ were studied using a Bruker spectrometer to obtain the FT-IR spectra within the 400–4000 cm^−1^ range in ATR mode. EDS-mapping were performed using HITACHI S-3400N.

The thermogravimetric analysis of the composite was carried out by differential scanning calorimeter DSC-600S. The sample to be tested was ground into powder to ensure sample uniformity and good thermal conductivity. A sample of about 10 mg was weighed and placed in a crucible. The test parameters of the equipment were set as nitrogen carrier gas, the flow rate was 20 ml min^−1^, the heating rate was 10 K min^−1^, and the pyrolysis temperature was 298–973 K. The crucible containing the sample was placed into the heating furnace of the thermogravimetric analyzer to begin the experimental procedure. The instrument automatically recorded the change of sample mass with temperature and generated a thermogravimetric curve (TGC).

### 2.6 Adsorption experiments

The static water vapor adsorption curves were carried out by gravimetric gas/vapor sorption analyzer (BSD-DVS). Firstly, the samples were dried thoroughly in a vacuum oven at 473 K for 12 h, and then transferred to the analysis station after weighing. The water vapor adsorption–desorption isotherm of the materials were measured at 298 K and 0–3.169 kPa pressure range, from which the maximum water vapor adsorption capacity of the material in the pressure range could be obtained.

The water vapor penetration adsorption curves were measured by multi-constituent adsorption breakthrough curve analyze (BSD-MAB). The inner diameter of the column was 0.6 cm, and the loading height of the sample was about 10 cm. Before the test, the sample was heated and purged with nitrogen for activation, with a heating temperature of 423 K and a heating time of 2 h. The test temperature was 298 K, with nitrogen as the carrier gas and a total flow of 300 sccm. The concentration and composition of gas at the outlet of the adsorption column were detected by an on-line mass spectrometer.

The adsorption–desorption curve of water vapor cycle was tested by dynamic gas/vapor sorption analyzer (BSD-DVS). The sample was degassed for 3 h at 573 K and transferred to the analysis station after weighing. Nitrogen was used as the carrier gas, with a total flow of 400 sccm. The isothermal adsorption–desorption curve of CaCl_2_/MOF-303 composite adsorbent was determined by 50 times of water vapor cycle at 298 K. The adsorption equilibrium condition was 0.1 mg/60 min, and the upper limit of the equilibrium time was 180 min. The degassing method was heating and atmospheric pressure purging.

## 3 Results and discussion

### 3.1 Synthesis and characterization

**Fig. 2:**
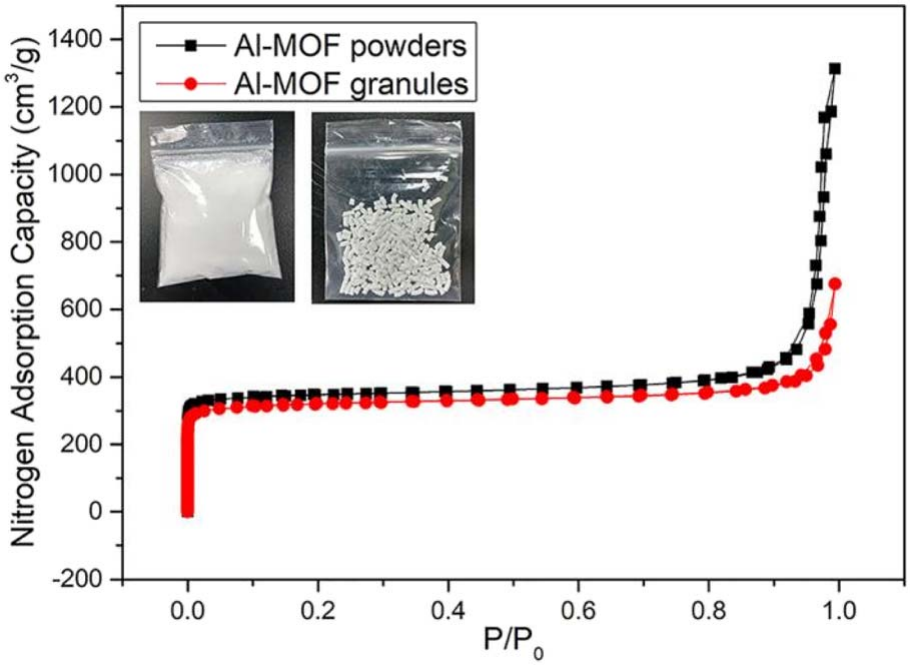
Nitrogen adsorption curves of MOF-303 before and after granulation.

In this study, a typical MOF-303 was selected as the experimental material. The adsorption isotherm of this MOFs was in the shape of steps, and the step points were located in low pressure area. The synthesis of MOF-303 was performed using the method mentioned in ref. 25, and the N_2_ adsorption isotherm at 77 K was shown in Fig. 2. As can be seen from the figure, the adsorption isotherm of MOF-303 first increased rapidly in the extremely low pressure region (*P*/*P*_0_ < 0.05), indicating the existence of a large number of micropores. When *P*/*P*_0_ ranged from 0.1 to 0.9, the nitrogen adsorption curve increased slowly and continuously. When *P*/*P*_0_ > 0.9, the nitrogen adsorption curve rose rapidly and a hysteresis loop appeared, indicating nitrogen condensation. The BET specific surface area was obtained by Brunauer–Emmett–Teller (BET) calculation method, and the BET specific surface area of the prepared MOF-303 was 1375 m^2^ g^−1^, which was basically consistent with the value in the literature.^29^ Using 5 wt% methylcellulose as the binder, the MOF-303 powder was extruded and granulated. The N_2_ adsorption isotherm of the MOF-303 granules at 77 K was shown in Fig. 2. It can be noted that compared with MOF-303 powder, the adsorption isotherm step point of MOF-303 granules showed a lag. The BET surface area of MOF-303 granules was 1276 m^2^ g^−1^, which was 7.2% lower than that of MOF-303 powder.

**Fig. 3:**
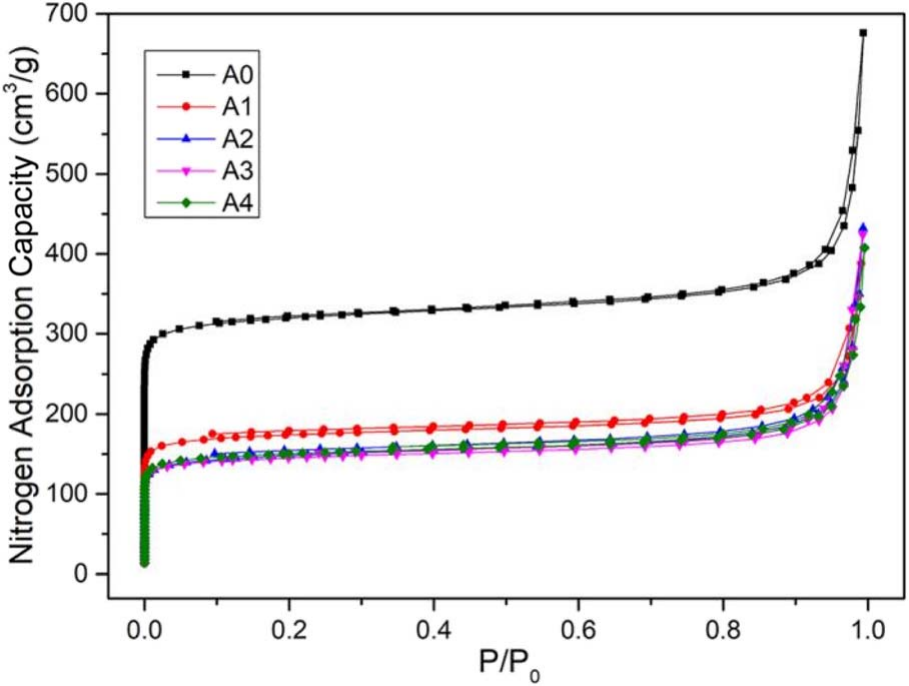
Nitrogen adsorption curves of MOF-303 and CaCl_2_/MOF-303 composites.

**Fig. 4:**
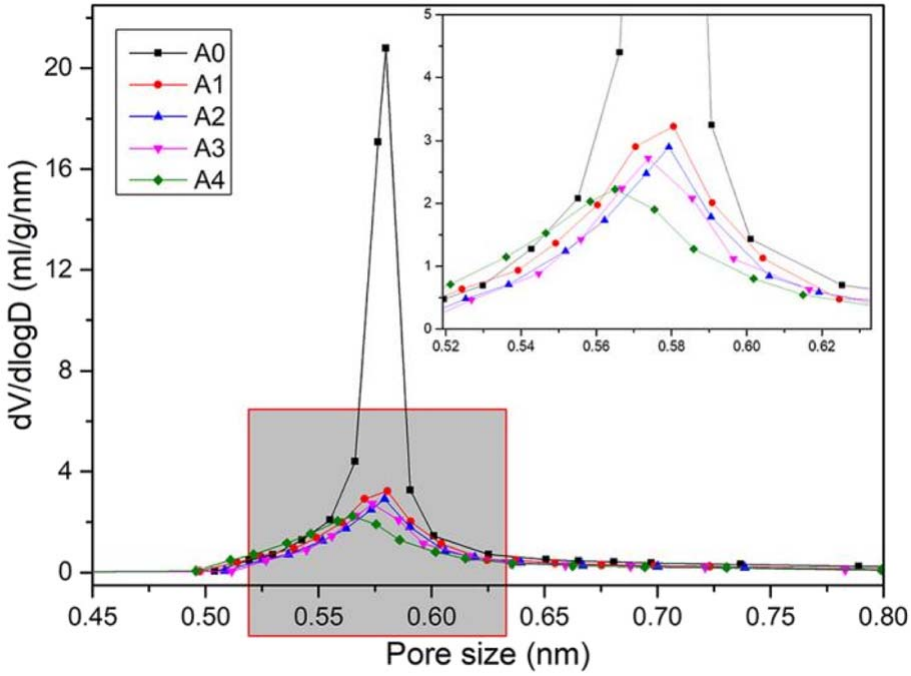
Pore size distribution curves of MOF-303 and CaCl_2_/MOF-303 composites.

CaCl_2_/MOF-303 composites were prepared by impregnating MOF-303 granules in CaCl_2_ solutions of different concentrations. MOF-303 was marked as A0, and CaCl_2_/MOF-303 composites were named A1, A2, A3 and A4 according to the corresponding impregnation concentration of CaCl_2_. The N_2_ adsorption isotherms of A0, A1, A2, A3 and A4 materials were shown in Fig. 3, showing the same adsorption trend. The pore size distribution curves based on H–K method was shown in Fig. 4. Table 1 listed the calcium content analysis, specific surface area, pore volumes and pore sizes for each material. The CaCl_2_ content of CaCl_2_/MOF-303 composites were 3.28–8.93 wt%. The BET specific surface area of CaCl_2_/MOF-303 composites were 568–683 m^2^ g^−1^, which was 45–55% lower than that of MOF-303. The results showed that the specific surface area and pore volume of the composites decreased with the increase of calcium chloride content, but the pore distribution trend remained unchanged, indicating that the impregnation salt blocked some pores, but did not destroy the overall structure of MOF-303.

**Table 1:** Pore structure and CaCl_2_ content of MOF-303 and composites

Materials	CaCl_2_ content^a^ (wt%)	Specific surface area^b^ (m^2^ g^−1^)	Pore volume (cm^3^ g^−1^)	Pore size^c^ (nm)
A0 powder	0	1375	1.012	0.586
A0 granule	0	1276	0.946	0.582
A1	3.28	683	0.604	0.581
A2	5.47	575	0.588	0.579
A3	8.66	568	0.547	0.576
A4	8.93	561	0.526	0.573

a The CaCl_2_ content values were determined by ICP-OES element analyzer.

b The specific surface area was calculated by BET method.

c The pore size distribution was analyzed by HK method.

In addition, in order to facilitate the comparison between the water adsorption properties of synthetic materials and traditional water absorption materials, the pore structure properties of 5A, 13X molecular sieve and A-type silica gel were tested, and their BET specific surface areas were between 465 and 596 m^2^ g^−1^.

**Fig. 5:**
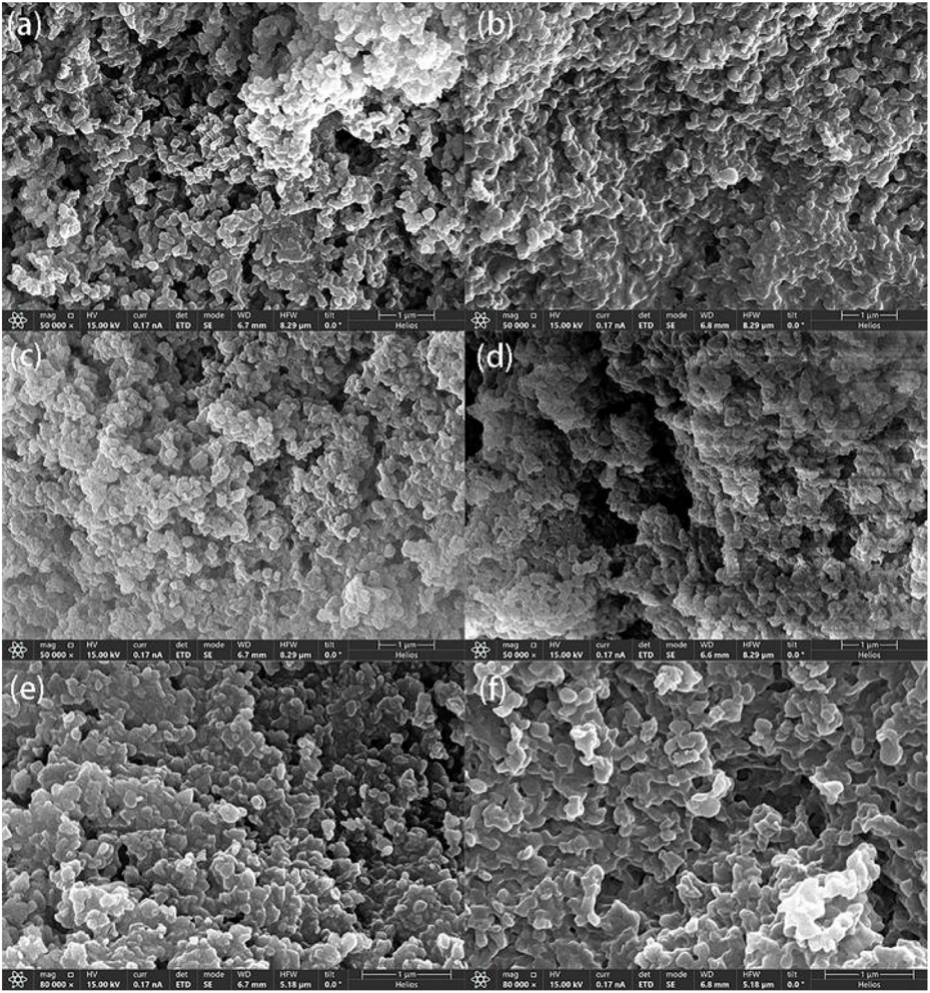
SEM images of (a) A1, (b) A2, (c) A3, (d) A4, (e) A0 and (f) A4 after washing.

**Fig. 6:**
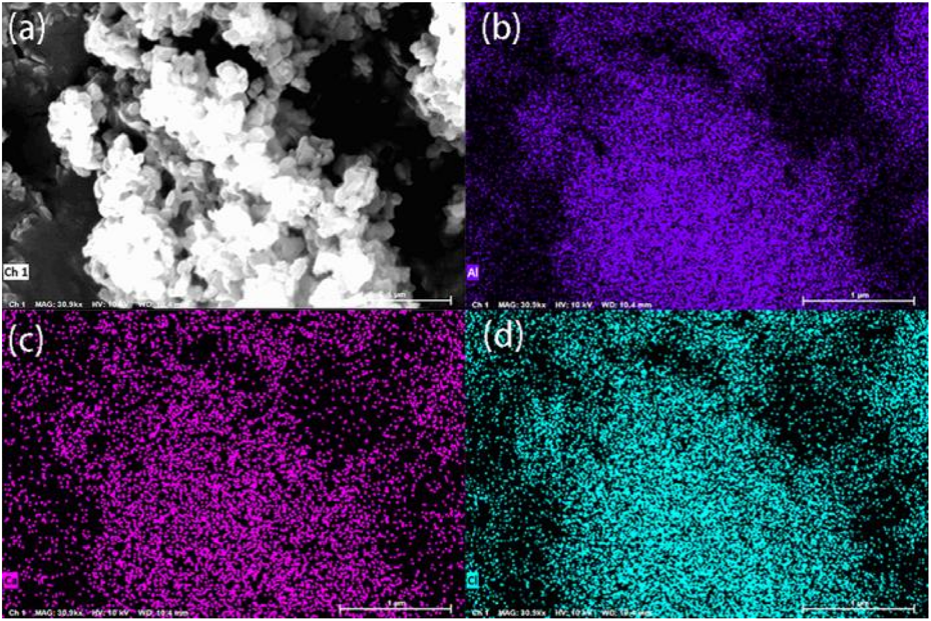
(a) Total, (b) Al, (c) Ca, and (d) Cl element distribution of CaCl_2_/MOF-303 composite.

The morphology of CaCl_2_/MOF-303 composites was studied by scanning electron microscopy (SEM), as seen in Fig. 5. With the increase of CaCl_2_ content, the morphology of the composites was also different. A1 showed a similar morphology to MOF-303. With the increase of CaCl_2_ loading, the degree of particle heterogeneity increased slightly, and even small aggregates were formed, indicating that CaCl_2_ particles were partially located in the intergranular pores of MOF-303. In A3 and A4, a large amount of CaCl_2_ was deposited on the surface of MOF-303 particles, resulting in forming large salt aggregates on the surface of MOF-303, which were unevenly distributed in the host material particles. This may be due to the location of the salt and diffusion being affected and the salt not being able to fully enter the MOF-303 pores. However, SEM micrographs of A4 after washing showed that the morphology of MOF-303 crystals did not change after CaCl_2_ loading, which also confirmed that the structure of the main material MOF-303 was not destroyed. Elemental mapping with energy dispersive X-ray spectrometry (EDS) was also performed, which revealed a highly uniform distribution of Al, Ca, and Cl atoms (Fig. 6).

**Fig. 7:**
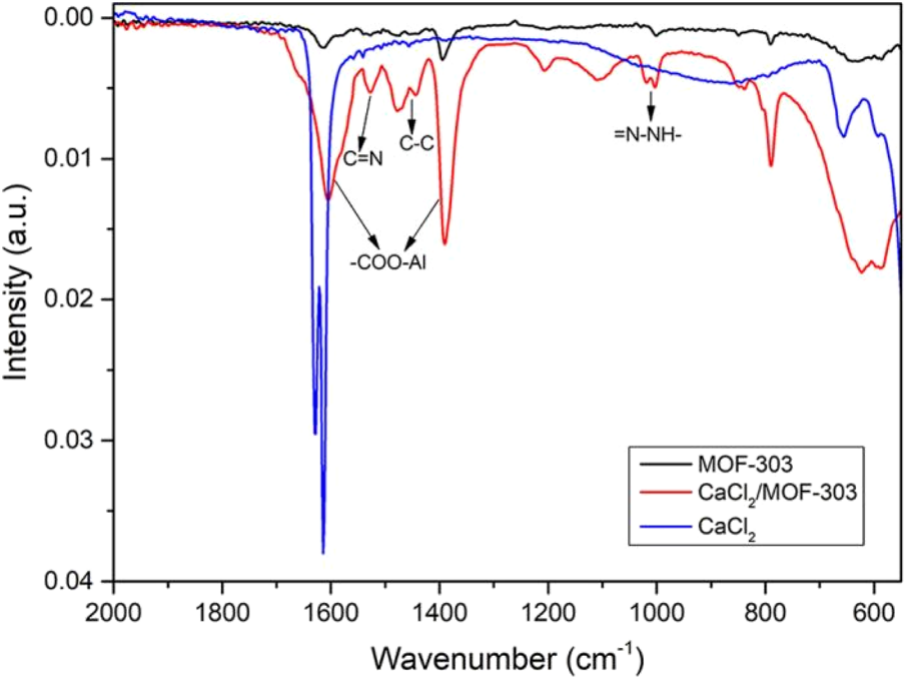
FTIR spectra of MOF-303, CaCl_2_/MOF-303 and CaCl_2_.

The chemical structure was verified by studying FTIR spectra of MOF-303, CaCl_2_/MOF-303 and CaCl_2_ (Fig. 7). The peaks at 1001 cm^−1^, 1478 cm^−1^, and 1529 cm^−1^ corresponded to the vibrations of =N–NH–, C–C, and C=N bonds on the pyrazole ligand, respectively. In addition, the peaks at 1386 cm^−1^ and 1604 cm^−1^ were attributed to the vibration of the –COO–Al bond, confirming the coordination between the Al^3+^ and H_3_PDC ligand, and the successful synthesis of MOF-303.

**Fig. 8:**
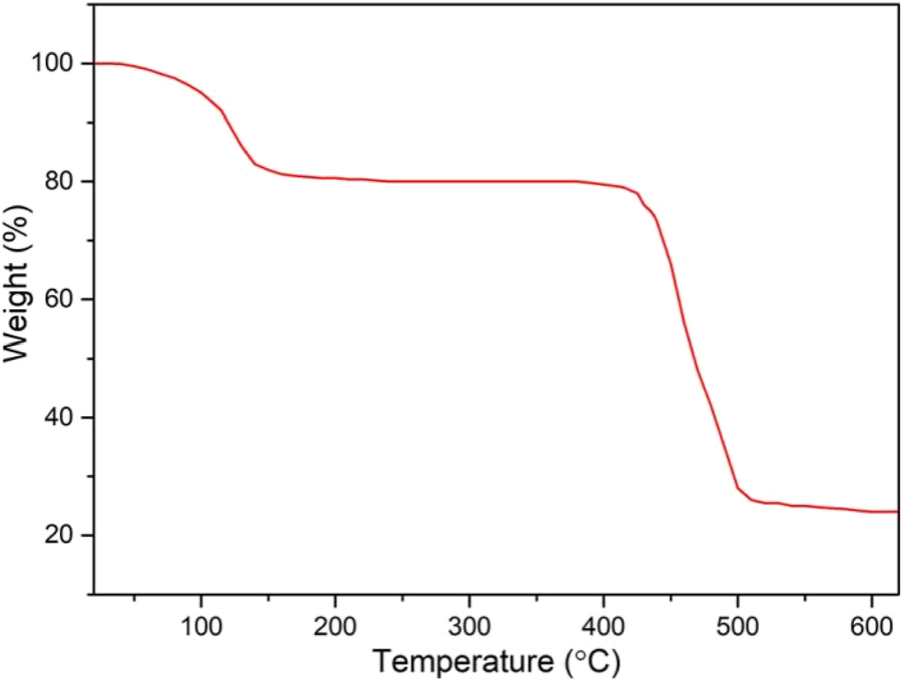
Thermogravimetric curve of the CaCl_2_/MOF-303 composite.

The thermogravimetric analysis of the CaCl_2_/MOF-303 composites was studied by heating them at a constant rate of 10 K min^−1^ and a flow rate of 20 ml min^−1^ under the condition of nitrogen flow at 298–973 K (Fig. 8). As shown in the figure, the TGA spectrum of the CaCl_2_/MOF-303 composite had two times of obvious weight loss in the range of 298–433 K and 673–773 K, corresponding to the evaporation of adsorbed water vapor and the pyrolysis of the composite material respectively.

### 3.2 Static water vapor adsorption

**Fig. 9:**
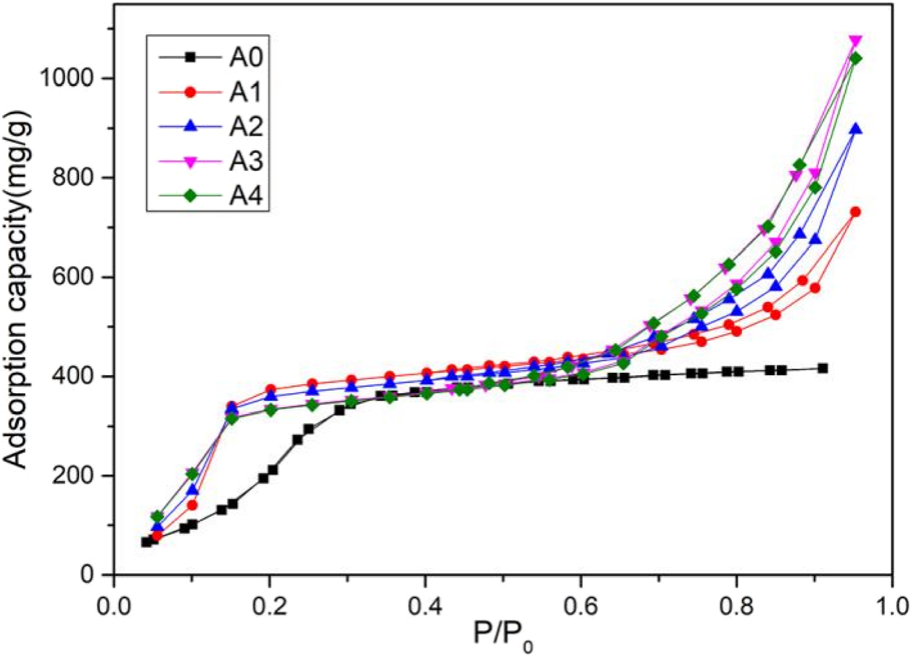
Water adsorption curves of MOF-303 and CaCl_2_/MOF-303 composites.

**Fig. 10:**
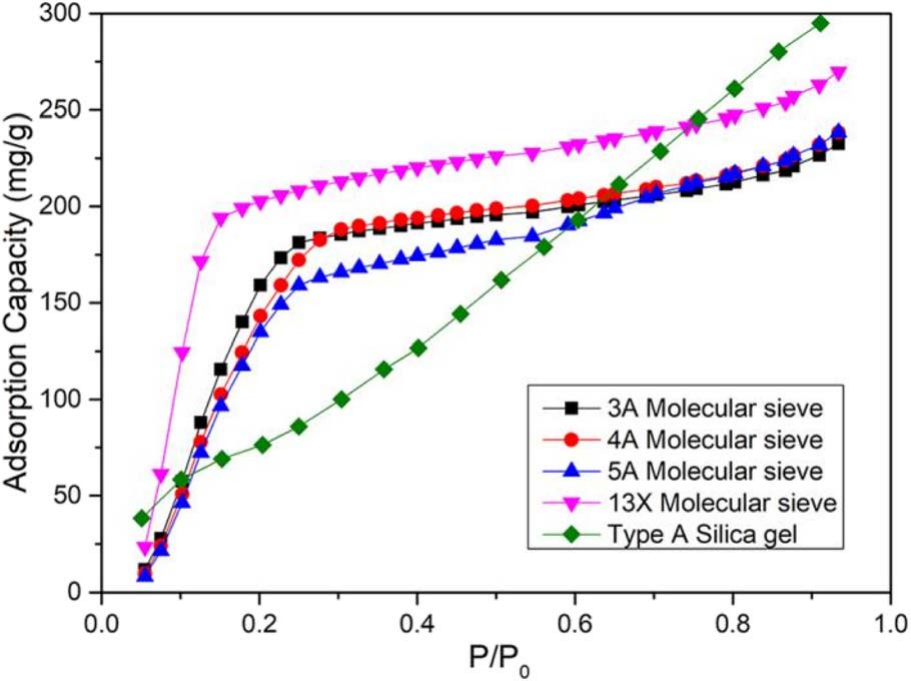
Water adsorption curves of traditional materials.

The static water vapor adsorption of MOF-303 and composite materials (A1, A2, A3 and A4), 3A, 4A, 5A, 13X microporous molecular sieve and A-type silica gel was studied by BSD-DVS dynamic gas/vapor sorption analyzer. The water vapor adsorption curves of MOF-303 and composite materials, molecular sieves and silica gel were shown in Fig. 9 and 10. It can be seen that the vapor adsorption isotherm of MOF-303 rose slowly at low pressure, and when *P*/*P*_0_ = 0.3, the adsorption isotherm rose gently, and the water vapor adsorption amount basically reached equilibrium. While the water vapor adsorption isotherms of the composites had three adsorption stages, which had the characteristics of S-type isotherm. When *P*/*P*_0_ < 0.15, the adsorption isotherms increased rapidly, and the adsorption capacity reached or even slightly exceeded the adsorption capacity of MOF-303, but the adsorption rate was much faster than that of MOF-303. When *P*/*P*_0_ = 0.15–0.7, the adsorption isotherms of composites rose gently. While the adsorption isotherms showed an inflection point at *P*/*P*_0_ = 0.7 and increased exponentially. With the increase of CaCl_2_ content, the rising rate of the curve was faster. Compared to MOF-303, these four composites had higher water adsorption capacities. The hygroscopic properties of these salt-deposited MOF-303, in addition to increasing water adsorption at low relative pressures, also increased water adsorption exponentially at high relative pressures. In general, the isotherms of the composites exhibited double adsorption characteristics of host MOF-303 substrate and CaCl_2_ deposited salt. It is worth noting that due to the adsorption properties of CaCl_2_, the adsorption and desorption curves of the composite materials had a certain hysteresis.

The adsorption capacities of MOF-303 and composite materials (A1, A2, A3 and A4), molecular sieve (3A, 4A, 5A and 13X) and A-type silica gel for water vapor were shown in Table 2. It can be seen from the data that the adsorption capacities of the molecular sieve materials for water vapor were concentrated in the range of 232–269 mg g^−1^. The adsorption capacity of 13X molecular sieve for water vapor was the highest, 269 mg g^−1^. The adsorption capacity of type A silica gel for water vapor was 295 mg g^−1^. The water vapor adsorption capacity of the synthesized MOF-303 powders was 445 mg g^−1^, and the water adsorption capacity of the prepared MOF-303 granules was 416 mg g^−1^, which reached more than 92% of that of the powders. The water adsorption capacities of CaCl_2_/MOF-303 composites were 731–1077 mg g^−1^, which was 76–159% higher than that of MOF-303, and 3.7–4.6 times that of traditional molecular sieves and silica gel. Among the CaCl_2_/MOF-303 composites, A3 had the highest water vapor adsorption capacity (1077 mg g^−1^).

**Table 2:** Water adsorption capacities of different materials

Materials	Water adsorption capacities (mg g^−1^)	Ref.
A0 powder	445	This work
A0 granule	416	This work
A1	731	This work
A2	897	This work
A3	1077	This work
A4	1040	This work
A1′	492.3	This work
A2′	523.9	This work
A3′	555.7	This work
A4′	562.2	This work
3A molecular sieve	232	This work
4A molecular sieve	238	This work
5A molecular sieve	238	This work
13X molecular sieve	269	This work
Type A silica gel	295	This work
UiO-66	360	31
UiO-66-NH_2_	370	31
UiO-66-NH_3_^+^Cl^−^	640	31
MIL-125	540	31
MIL-125-NH_2_	530	31
MIL-125-NH_3_^+^Cl^−^	590	31
NH_2_-MIL-125	420	32
CPO-27(Ni)	410	33
MIL-100(Al)-GO	526–606	34
CAU-10-H-GO	272–350	34
CaCl_2_@UiO-66_53	600	35
Aluminium fumarate-CaCl_2_	680	36

The CaCl_2_/MOF-303 composite adsorbents retained the micropore characteristics of MOF-303 to a large extent, and still had developed pores, giving full play to the synergistic effect of physical adsorption and chemical adsorption of MOF-303 carrier and CaCl_2_ deposited salt, and enhancing the adsorption performance. However, the impregnation of CaCl_2_ decreased the pore volume of the adsorbents and increased the diffusion resistance of water molecules in the process of entering the pore of the adsorbents. Therefore, with the increase of impregnation amount, the permeability and adsorption capacity of the composite adsorbent first increased and then decreased under the opposite effect of chemisorption enhancement and adsorbent pore volume reduction.

In addition, the water adsorption properties of CaCl_2_/MOF-303 composite prepared by impregnation method and simple physical mixing were also compared. According to the elemental analysis results of the composite prepared by impregnation method, CaCl_2_ powder and MOF-303 powder were mixed according to the content of CaCl_2_, and their water adsorption properties were tested (Table 2). Compared with the physical mixing method, the impregnation method can more effectively disperse CaCl_2_ evenly in the pores of MOF-303, while the physical mixing can only mix the powder, unable to achieve such deep dispersion, and it is difficult to directly apply to the actual scene. The test results show that the water adsorption properties of the composite prepared by physical mixing method are obviously lower than those prepared by immersion method. This result further demonstrates the superiority of the impregnation method in the dispersion uniformity of CaCl_2_ and the synergistic effect between CaCl_2_ and MOF-303 achieved by the impregnation method.

In the reported literature on MOFs used for dehumidification, the water adsorption capacities of UiO-66 series were 360–640 mg g^−1^,^31^ MIL-125 series were 420–590 mg g^−1^,^31,32^ and CPO-27(Ni) was 410 mg g^−1^.^33^ The water adsorption capacities of GO/MOFs such as CAU-10-H-GO and MIL-100(Al)-GO were 272–606 mg g^−1^.^34^ While the water adsorption capacities of other MOFs modified by CaCl_2_ such as CaCl_2_@UiO-66_53 and aluminium fumarate–CaCl_2_ were 600–680 mg g^−1^.^35,36^ The water adsorption capacities of CaCl_2_/MOF-303 composites prepared in this work was much higher than that of other MOFs as reported, showing great potential in dehumidification applications.

### 3.3 Dynamic water vapor adsorption

**Fig. 11:**
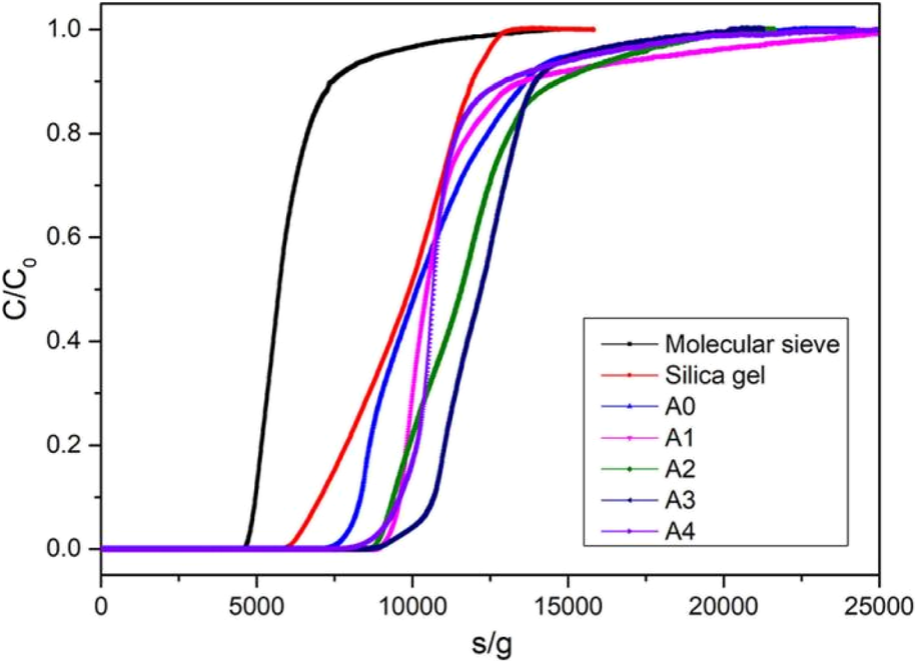
Penetration curves of water vapor on different materials.

The water vapor breakthrough adsorption curves of materials were determined by BSD-MAB multi-constituent adsorption breakthrough curve analyzer. The water vapor dynamic adsorption properties of MOF-303 and composites (A1, A2, A3 and A4), microporous molecular sieve and A-type silica gel at 298 K and atmospheric pressure were compared. The breakthrough adsorption curves of water vapor on each material were shown in Fig. 11, and the breakthrough adsorption capacities and equilibrium adsorption capacities of each adsorbent were shown in Table 3. It can be seen from the curves that water vapor quickly penetrated and reached adsorption equilibrium on the molecular sieve. The water vapor breakthrough adsorption capacity and equilibrium adsorption capacity of MOF-303 and composites were higher than those of molecular sieve and silica gel. The water vapor dynamic permeation adsorption capacities of MOF-303, molecular sieve and silica gel were 248.6 mg g^−1^, 151.2 mg g^−1^ and 207.0 mg g^−1^, respectively. With the increase of calcium chloride content, the dynamic equilibrium adsorption amount of the composite adsorbent increased significantly. The water vapor dynamic permeability adsorption capacities of CaCl_2_/MOF-303 composites were 277.2–300.6 mg g^−1^, among which A3 had the highest dynamic permeability adsorption capacity (300.6 mg g^−1^) and the longest permeability time. The result was consistent with the trend of static adsorption of water vapor of CaCl_2_/MOF-303 composites, that was, there was an optimal amount of CaCl_2_ content. The adsorption breakthrough time increased with the increase of CaCl_2_ content in the composites. When the CaCl_2_ content increased further, the pore plugging of the composite would be aggravated, and the adsorption breakthrough time would decrease.

**Table 3:** Water breakthrough adsorption capacity and breakthrough time of each material

Materials	Breakthrough adsorption capacity (mg g^−1^)	Breakthrough time(min g^−1^)
A0	248.6	134.3
A1	277.2	152.3
A2	288.0	153.1
A3	300.6	169.2
A4	293.4	156.9
13X molecular sieve	151.2	80.8
Type A silica gel	207.0	110.0

**Fig. 12:**
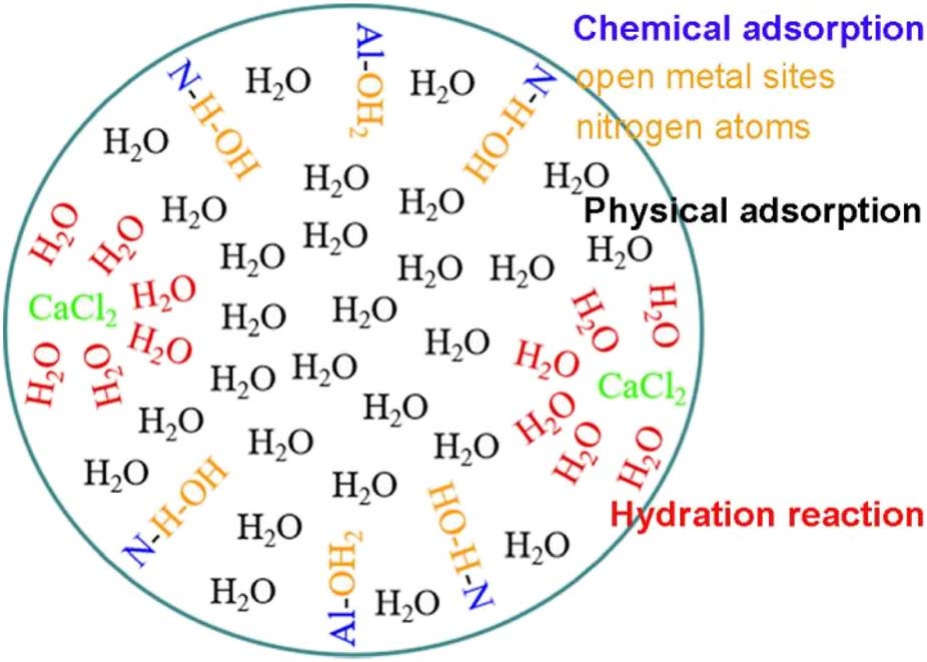
Schematic diagram of water adsorption mechanism of CaCl_2_/MOF-303 composites.

There are three main mechanisms for the adsorption of water vapor by CaCl_2_/MOF-303 composites (Fig. 12): firstly, the strong affinity of water to open metal sites on MOF-303 and nitrogen atoms on organic ligands caused chemical adsorption. The second was the physical adsorption of water in MOF-303 micropores. Water molecules were first adsorbed by open metal sites and nitrogen atoms on MOF-303, and then attracted more water molecules by forming hydrogen bonds, thus forming water clusters. Lastly, the water adsorption mechanism of CaCl_2_ in composites was realized through hydration reaction, in which water molecules would hydrate with CaCl_2_ molecules in the composites to form CaCl_2_ hydrate, thereby absorbing a large amount of water.

### 3.4 Water vapor cycle adsorption and desorption

**Fig. 13:**
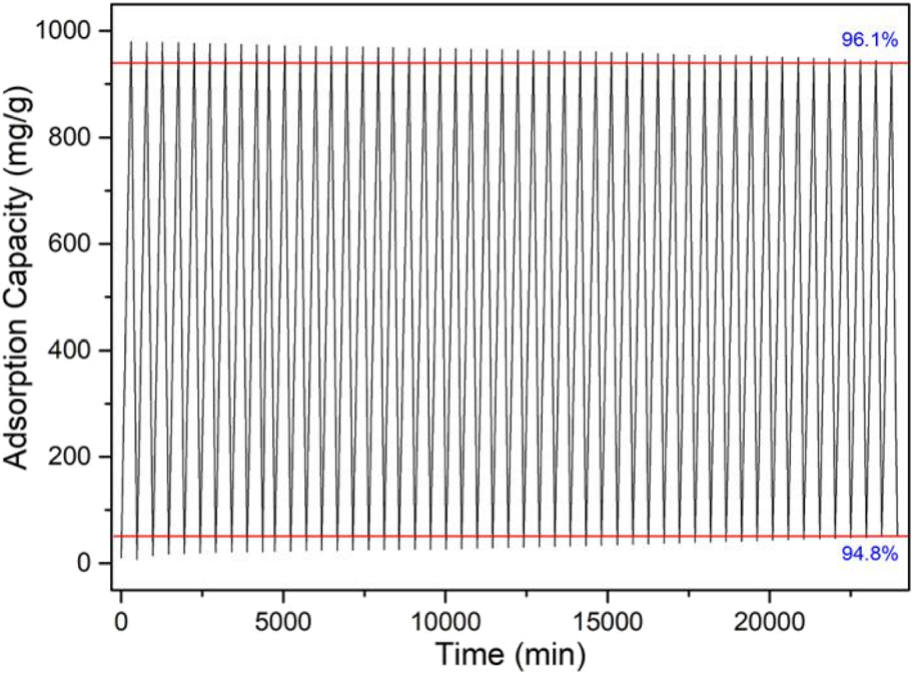
Cyclic adsorption and desorption of CaCl_2_/MOF-303 (3).

The regeneration performance of materials is the key factor to evaluate whether it has practical application value. In order to investigate the regeneration ability and stability of the CaCl_2_/MOF-303 composites, 50 consecutive water vapor adsorption and desorption cycles were carried out with the BSD-DVS dynamic gas/vapor sorption analyzer. The cyclic adsorption and desorption curves of water vapor on CaCl_2_/MOF-303 composite was shown in Fig. 13. The maximum adsorption capacity of water vapor on the CaCl_2_/MOF-303 composite after 50 cycles of water vapor adsorption–desorption did not change significantly, the retention rate was 96.1%, and the desorption rate decreased slightly to 94.8%. The above data indicated that the CaCl_2_/MOF-303 composite is a kind of excellent reusable adsorbent with high water vapor adsorption capacity, fast adsorption rate, good regeneration performance and high structural stability.

In addition, in order to evaluate the stability of CaCl_2_ in the adsorption–desorption cycle of composite materials, we detected the content of CaCl_2_ in the adsorbent after recycling by elemental analysis to determine whether CaCl_2_ leakage occurred. The experimental results showed that the content of CaCl_2_ in the composite was basically consistent with that before use after 50 adsorption–desorption cycles. This indicates that CaCl_2_ has excellent dispersion and fixability in the pore structure of MOF-303, which can effectively prevent the leaching of CaCl_2_ during the cycle. This structural advantage not only ensures that the CaCl_2_/MOF-303 composite maintains a high adsorption performance in multiple cycles, but also significantly improves its structural stability. Based on the above experimental results, we believe that CaCl_2_/MOF-303 composites show great application potential in dehumidification and other related fields due to its excellent salt leakage resistance and high regeneration efficiency.

## 4 Conclusions

In this study, CaCl_2_/MOF-303 granule composite adsorbents were prepared by an impregnation method. The composite adsorbent showed the following characteristics:

(1) Under the synergistic action of physical adsorption and chemical adsorption, the water adsorption effect of composite adsorbents is obviously better than that of traditional water adsorption materials such as molecular sieves. The water adsorption capacities of CaCl_2_/MOF-303 composites were 731–1077 mg g^−1^, which was 76–159% higher than that of MOF-303 without CaCl_2_, and 3.7–4.6 times that of molecular sieves and silica gel, and 1.6–4.0 times that of the reported values of MOFs.

(2) The water adsorption performance of CaCl_2_/MOF-303 composites is related to the impregnation amount of CaCl_2_, and the equilibrium adsorption capacity and breakthrough adsorption capacity all had a trend of first increasing and subsequent decreasing under the opposite effect of chemisorption enhancement and adsorbent pore volume reduction.

(3) After 50 water adsorption and dehydration cycles, the regenerative rate of the CaCl_2_/MOF-303 composite was 96.1%.

Therefore, the CaCl_2_/MOF-303 composite adsorbent has high water adsorption capacity, good dehydration properties and structural cycle stability, and is a kind of reusable water adsorption material with superior performance.

## Data availability

The data that support the findings of this study are available within the article.

## Author contributions

Dr Y. Li proposed the experimental ideas and wrote the manuscript. J. Li, S. Yin and X. Shan were responsible for the investigation and data analysis. B. Tao and S. Wang were responsible for the format modification and inspection of the paper.

## Conflicts of interest

There are no conflicts to declare.
